# 1535. Persistence With Human Immunodeficiency Virus Pre-exposure Prophylaxis in an Active-Duty Military Population

**DOI:** 10.1093/ofid/ofad500.1370

**Published:** 2023-11-27

**Authors:** Jacob T Colver, Joseph Yabes, Joseph Marcus

**Affiliations:** San Antonio Uniformed Services Health Education Consortium, San Antonio, Texas; Brooke Army Medical Center, San Antonio, Texas; Brooke Army Medical Center, San Antonio, Texas

## Abstract

**Background:**

There is limited data on Human Immunodeficiency Virus Pre-exposure Prophylaxis (PrEP) use and persistence in the military. Despite universal access to care, there is concern that PrEP persistence may be lower in military populations due to frequent deployments, need for relocations, and perceived stigma. This study evaluated the persistence rates as well as reasons for PrEP discontinuation in a military cohort.

**Methods:**

The Brooke Army Medical Center Infectious Diseases clinic provides PrEP to service members at Joint Base San Antonio. This study evaluated all active-duty service members who received PrEP between 2020-2022. All charts were examined to determine patient demographics and years on PrEP. If a patient stopped PrEP, they were contacted to determine etiology of discontinuation and invited to restart PrEP.

**Results:**

In total, 112 service members received PrEP during the study period. The cohort was predominantly male (99%) with median age 30 [IQR: 26-34] and a median of 2 years [IQR: 0-3] receiving PrEP (**Table 1**). The most common indication was multiple sexual partners with less than 100% condom use (n=98, 88%). At the end of the study, most (n=88, 79%) patients were still receiving PrEP including 33 (37%) at other military or civilian facilities. Of the 24 service members who were no longer receiving PrEP, 18 (75%), were able to be contacted (**Table 2**). Two (1%) patients seroconverted while off PrEP with reasons for discontinuation being abstinence and difficulty making appointments. No patients contacted during this study were interested in restarting PrEP.

Demographic Information of 112 Active-Duty Service Members Receiving PrEP at Brooke Army Medical Center 2020-2022

**Figure:**
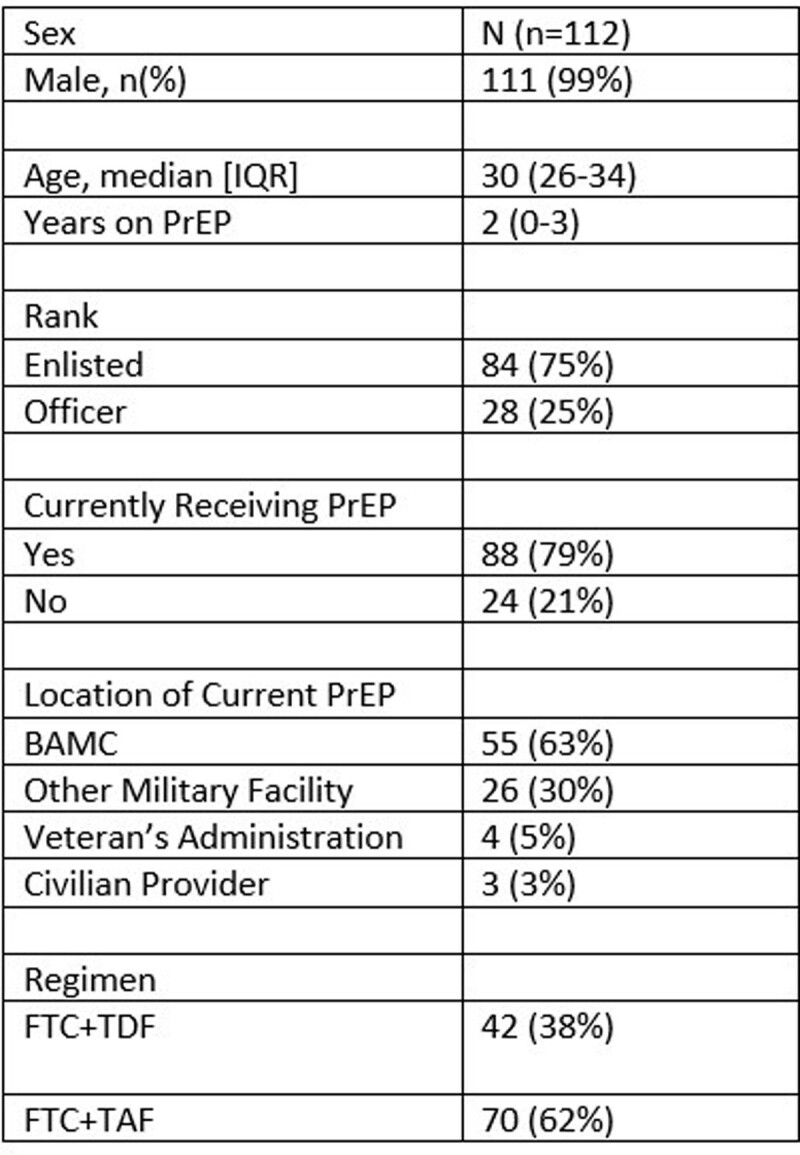

Reasons for PrEP Discontinuation at Brooke Army Medical Center 2020-2022

**Figure:**
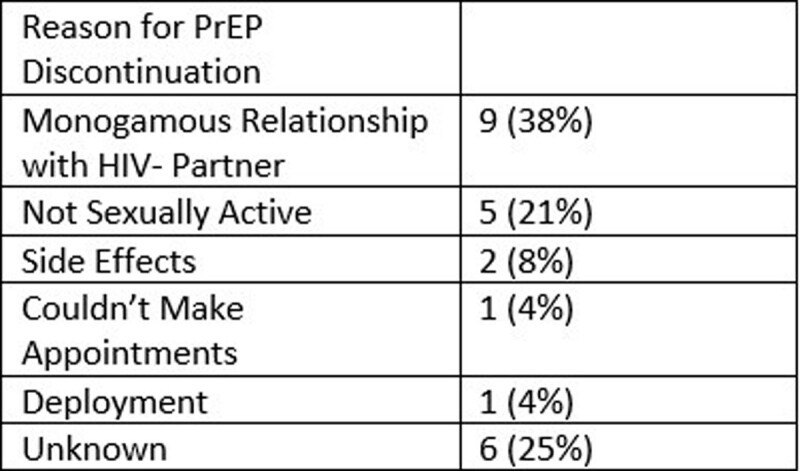

**Conclusion:**

This is the first study to evaluate PrEP persistence in an active-duty population. In this cohort with universal access to care, PrEP persistence rates and years receiving PrEP were greater than seen in other populations. While the most common reason for discontinuation was changes in sexual behavior, systemic factors still contributed to PrEP discontinuation. Future studies should elucidate the challenges to PrEP care in the military.

**Disclosures:**

**All Authors**: No reported disclosures

